# Meeting technical challenges for protein characterization and surrogate equivalence studies that resulted from insecticidal protein co-expression in maize event MZIR098

**DOI:** 10.1007/s11248-019-00183-w

**Published:** 2019-11-28

**Authors:** Frederick S. Walters, Scott Young, Gerson Graser

**Affiliations:** Syngenta Crop Protection, LLC, 9 Davis Drive, P.O. Box 12257, Durham, NC 27709 USA

**Keywords:** Insecticidal, Cry protein, Rootworm, Transgenic, Equivalence, Peptide mass coverage, Bioassay

## Abstract

Safety assessment of genetically modified plants includes protein characterization to confirm the intended trait protein expression. In addition, to conduct safety tests, the large amount of purified protein needed is usually met through the use of a surrogate, microbially produced protein source. Characterization of the eCry3.1Ab and mCry3A proteins as derived from Event MZIR098 maize was challenging because of the difficulty in purifying/isolating these proteins that are of similar molecular weight and have considerable shared sequence and immunogenicity. This also applies to establishing the biochemical equivalence to the microbially produced surrogate proteins, as highly-purified plant protein is required. While use of crude plant extracts facilitated functional equivalence testing with the surrogate proteins, a separate technical challenge had to be met. The eCry3.1Ab and mCry3A proteins display differentiated modes of action toward CRW pests, however, with the same overall target pest spectrum, no differential test organism existed to allow equivalence testing for one insecticidal protein in the presence of the other. To establish that the microbially produced proteins are suitable surrogates for the plant-produced proteins, the challenges in the protein purification and bioactivity testing had to be addressed. This article describes technical solutions to assess and characterize the insecticidal proteins in this new event and thereby confirm equivalence/suitability of the microbially produced protein surrogates.

**Electronic supplementary material:**

The online version of this article (10.1007/s11248-019-00183-w) contains supplementary material, which is available to authorized users.

## Introduction

Syngenta transformed maize (*Zea mays* L., corn) to produce Event MZIR098 maize, which provides dual modes of action in consideration of Insect Resistance Management (IRM), for controlling three of the major corn rootworm (CRW) pests in North America: *D. virgifera virgifera* (western corn rootworm), *D. longicornis barberi* Smith and Lawrence (northern corn rootworm), and *D. virgifera zeae* Krysan and Smith (Mexican corn rootworm). Although none of the current CRW control traits are high dose and the potential for field-derived cross-resistance amongst these traits has been noted (Jakka et al. 2016; Wen and Chen 2018), IRM best practices for CRW control do not rely on a singular technology (such as transgenic crop deployment), or single-year approach. Effective CRW management should be designed at the individual field level for multiple years ahead, integrating differentiating control measures including crop rotation, trait technology, soil-applied insecticides, and adult beetle control (Syngenta 2018).

MZIR098 maize plants contain the transgenes *ecry3.1Ab* and *mcry3A*, encoding the proteins eCry3.1Ab and mCry3A (NCBI accession numbers GU327680 and AX712174), which are identical to the insecticidal proteins produced by Syngenta Event 5307, and Event MIR604 maize, respectively. Detailed molecular characterization of the MZIR098 insert sequence elements confirmed that only the intended *ecry3.1Ab*, *mcry3A*, and *pat* (phosphinothricin acetyltransferase, PAT) transgenes were present (FSANZ 2016). PAT confers tolerance to glufosinate-ammonium in herbicide products (Hérouet et al. 2005) and was used as a selectable marker in the development of MZIR098 maize. The eCry3.1Ab and mCry3A insecticidal proteins have been demonstrated to be safe for humans, livestock, and the environment (US EPA 2007a, b, 2012a, b; Raybould et al. 2007; Burns and Raybould 2014) and are already available in combination in the Agrisure Duracade™ maize breeding-stack. By combining these insecticidal protein traits at a single breeding locus (in MZIR098), the efficiency of trait conversion into elite genetic lines can be increased, thus improving the ability for other traits to be combined in commercial maize products to meet grower needs. Since event MZIR098 maize was made through a new transformation, the EPA presently requires a full product characterization of each plant incorporated protectant (PIP), including protein characterization and expression, biochemical characterization and examination of any post-translational modifications of expressed substance(s) of the inserted PIP trait, and characterization of any surrogate test substance derived from an alternative expression system (US EPA 2016). The combination of the two insecticidal trait proteins in a single event introduced unique technical challenges for characterization in comparison to having the proteins in separate events.

The purpose of this present study, therefore, as part of safety assessment for plants genetically modified to express trait protein, was two-fold: (1) to characterize and confirm the intended expression of the insecticidal proteins in plants derived from Event MZIR098, and (2) to demonstrate that microbially produced eCry3.1Ab and mCry3A could be used as protein surrogates in safety studies, which require large amounts of test material. Characterization of the eCry3.1Ab and mCry3A plant-produced proteins was problematic because of the difficulty in purifying/isolating these two proteins that are of similar molecular weight and have considerable shared sequence and immunogenicity. The use of microbially produced test substances are necessary for safety assessments of transgenic crops because some safety assessments require large amounts of protein, and it is often impractical to extract and purify the plant-produced protein in quantities sufficient for all studies (Raybould et al. 2013). To adopt this surrogate protein strategy, however, the biochemical and functional equivalence of the respectively-sourced proteins must be established. In the case of Event MZIR098, the aforementioned characterization challenge for the plant-produced proteins also applies for the biochemical equivalence testing where highly-purified plant protein is routinely used. In contrast, freshly-made crude plant extracts can be considered preferable to use in functional equivalence testing (as opposed to using stored, plant-purified protein) to avoid any protein modification or potential loss of activity which could occur during purification steps and extended exposure to the disrupted plant matrix (Doran 2006; Jervis and Pierpoint 1989; Wilken and Nikolov 2012).

A technical challenge existed for the functional equivalence testing in terms of making a bioactivity comparison of the microbially produced insecticidal proteins with the plant-produced proteins. This challenge was present as both eCry3.1Ab and mCry3A proteins targeted the same test insect, therefore no differential test organism existed to allow equivalence testing for one protein alone in the presence of the other protein.

Novel technical strategies met these challenges for the plant-purified protein characterization and biochemical equivalence testing needs (*e.g.*, SDS-PAGE electroelution to provide plant-purified proteins for Western blot, peptide mass coverage, and glycosylation blot analyses), and for the functional equivalence testing (*e.g.*, establishing the ratio of plant-produced insecticidal protein and matching that ratio for bioactivity assessment of a combination of microbially produced insecticidal proteins). Here, we describe these innovative approaches which were developed to characterize the MZIR098 plant-produced insecticidal proteins and to confirm equivalence of the heterologously produced protein surrogates which are used in studies to establish the safety of the transgenic maize event.

## Materials and methods

### Microbially produced insecticidal proteins

The microbially produced eCry3.1Ab and mCry3A proteins were each prepared from *Escherichia coli* expression systems, purified by liquid chromatography, and processed into respective lyophilized powders. The preparations were determined to contain 87.8% eCry3.1Ab and 75.0% mCry3A by weight, respectively. The amino acid sequence of the microbially produced eCry3.1Ab is the same as eCry3.1Ab expressed in Syngenta’s transgenic maize, Event MZIR098, except that the microbially produced protein contains one additional methionine and six additional histidine residues at the N-terminus. The additional amino acids (histidine tag) facilitated purification of the recombinant protein after production. The amino acid sequence of the microbially produced mCry3A is the same as mCry3A expressed in the Event MZIR098 maize. The microbially produced protein preparations were stored at – 20 °C ± 8 °C until further use.

### Plant material

Seed for the maize transgenic event and the near-isogenic, nontransgenic control materials were confirmed by real-time polymerase chain reaction (PCR) testing (Ingham et al. 2001) for identity. Plants were grown under standard greenhouse conditions, and leaf material was collected 4–6 weeks after emergence, frozen at − 80 °C ± 10 °C, ground into a fine powder, lyophilized and stored at − 80 °C ± 10 °C until further use. Identification of the transgenic and nontransgenic control leaf material was also verified by real-time PCR for Stewardship Quality Control testing.

### Plant crude extracts

Crude extracts of MZIR098 and nontransgenic maize leaf material were prepared by resuspending lyophilized maize leaf powder in phosphate buffered saline, pH 7.4 with 0.05% Tween® 20 (PBST) supplemented with two Complete® protease inhibitor cocktail tablets per 50 ml of buffer (Roche Diagnostics, Mannheim, DE). Each mixture was homogenized (Omni International), filtered across a 40 µM nylon cell strainer (500 × *g* for 1 min at 4 °C) and the filtrate was again centrifuged at 3000 × *g* for 15 min at 4 °C (Sorvall Legend RT). The resulting supernatants were stored on ice until use. The respective crude extracts of MZIR098 and nontransgenic maize leaf material were quantified (as described in sections below), detected by Western blot analysis and activity determined by insecticidal bioassay.

### Plant purified preparations

Several analyses also required further purification of plant-produced eCry3.1Ab and mCry3A from the crude extract of MZIR098 maize leaf tissue (e.g., to address any potential plant matrix effect on subsequent Western blot mobility, and to avoid cross-contamination of plant glycosylated protein for glycosylation blot analysis); therefore, eCry3.1Ab and mCry3A were each extracted in 100 mM sodium borate, 1.2% HCl, pH 7.5 buffer supplemented with 7.69 mM sodium azide in preparation for an initial immunoaffinity purification step. After homogenization, the extract was centrifuged for 20 min at 12,000 revolutions per minute (rpm) and filtered through a 0.2 μm Nalgene filter unit.

An immunoaffinity column, prepared with anti-mCry3A monoclonal antibodies, was used to simultaneously purify both eCry3.1Ab and mCry3A from the MZIR098 maize extract. The MZIR098 maize extract was applied to the equilibrated immunoaffinity column, the column was washed to remove unbound proteins, and both eCry3.1Ab and mCry3A were eluted in 100 mM glycine buffer (pH 2.5) and neutralized with 0.5 M sodium phosphate (pH 9.0). Fractions containing eCry3.1Ab and mCry3A were prepared in NuPAGE® lithium dodecyl sulfate (LDS) sample buffer and NuPAGE® reducing agent (Life Technologies, Carlsbad, CA, US) and further purified by resolving them in a preparative sodium dodecyl sulfate polyacrylamide gel electrophoresis (SDS-PAGE). The molecular weight standard was SeeBlue® Plus2 pre-stained standard (Life Technologies). All samples were subjected to SDS-PAGE under reducing conditions with a NuPAGE® 4–12% bis(2-hydroxyethyl)imino-tris(hydroxymethyl)methane (Bis–Tris) polyacrylamide gradient gel (Life Technologies) using 4-morpholinoepropanesulfonic acid (MOPS) running buffer (Life Technologies). The protein bands corresponding to eCry3.1Ab and mCry3A were excised independently, and electro-eluted per manufacturer’s instructions (Model 422 Electro-Eluter, Bio-Rad Laboratories, Hercules, CA, USA) in 50 mM ammonium bicarbonate, 0.1% SDS buffer, followed by dialysis in 50 mM ammonium bicarbonate, and concentration using a CentriVap® (Model 7,310,020, Labconco corp., Kansas City, MO) at 4 °C. These purified preparations of eCry3.1Ab and mCry3A from MZIR098 maize were used for Western blot and glycosylation analyses, and in-solution protein digestion in preparation for peptide mass coverage analysis, as described in respective sections below.

The various preparations of the insecticidal proteins and their use in subsequent analyses are summarized in Table 1 below.

**Table 1:** Insecticidal protein preparations and use in subsequent analyses

Analysis	Preparations included in the analysis	Purpose of the analysis
Western blot	Microbially produced eCry3.1Ab Microbially produced mCry3A MZIR098 maize crude extract Purified preparation of eCry3.1Ab from MZIR098 maize crude extract Purified preparation of mCry3A from MZIR098 maize crude extract Nontransgenic crude extract fortified with microbially produced eCry3.1Ab Nontransgenic maize crude extract fortified with microbially produced mCry3A	Examine eCry3.1Ab and mCry3A apparent molecular weight, intactness, and relative immunoreactivity
Peptide mass coverage	Microbially produced eCry3.1Ab, or mCry3A Purified preparation of eCry3.1Ab, or mCry3A from MZIR098 extract	Confirm the identity of both sources of protein Confirm the N-terminal amino acid sequence of both sources of protein
Glycosylation blot	Microbially produced eCry3.1Ab, or mCry3A Purified preparation of eCry3.1Ab, or mCry3A from MZIR098 extract	Confirm the absence of glycosyl residues
Insecticidal activity	Mixture of microbially produced eCry3.1Ab and mCry3A MZIR098 maize crude extract Nontransgenic maize crude extract fortified with a mixture of microbially produced eCry3.1Ab and mCry3A Nontransgenic maize crude extract	Confirm functional equivalence of microbial protein mixture with plant derived protein mixture

### Quantitation of total protein

Total protein in MZIR098 maize and nontransgenic maize crude extracts was quantitated via the bicinchoninic acid (BCA) method (Hill and Straka 1988), using bovine serum albumin as the reference protein standard. The results were analyzed with SoftMax Pro® GxP software, version 5.4.1 (Molecular Devices, LLC, San Jose, CA, US) using a four-parameter algorithm. For each sample, the mean concentration of all dilutions within the quantitative range of the BCA assay was calculated. An average amount of total protein from six independent extracts was determined for both MZIR098 maize and nontransgenic maize crude leaf extracts, respectively. Based on these results, the amount of total protein used in the Western blot analysis and in the insecticidal bioassay remained consistent between MZIR098 maize extract and fortified or non-fortified nontransgenic maize extract samples, where applicable.

### Quantitation of trait protein by ELISA

The concentration of eCry3.1Ab and mCry3A in the MZIR098 maize crude extract was quantified by ELISA (Tijssen 1985) to estimate concentrations for Western blot analysis and insecticidal activity bioassay. Quantification of eCry3.1Ab was performed using the Beacon Analytical Systems eCry3.1Ab ELISA double-antibody sandwich kit for eCry3.1Ab. The eCry3.1Ab protein was captured between an immobilized monoclonal antibody and a polyclonal rabbit antibody, both capable of binding to eCry3.1Ab. The eCry3.1Ab was detected by a secondary polyclonal antibody (donkey anti-rabbit immunoglobulin G, Jackson ImmunoResearch Laboratories, Inc., West Grove, PA, US) conjugated to alkaline phosphatase, which catalyzed the conversion of a colorimetric substrate.

Quantification of mCry3A was performed using the Envirologix Qualiplate™ ELISA Kit for mCry3A. The mCry3A was captured on ELISA plates pre-coated with the capture antibody. An antibody-enzyme conjugate was used to bind the mCry3A protein and detection was accomplished through conversion of a colorimetric substrate.

For both eCry3.1Ab and mCry3A ELISA, the concentration of protein was proportional to the measured absorbance values. Samples were quantified relative to a standard curve of known respective insecticidal protein concentrations. Samples and standards were applied to the microtiter plates in triplicate. The absorbance values were measured with a spectrophotometer at dual wavelengths of 405 and 490 nm or a single wavelength of 450 nm, for eCry3.1Ab, or mCry3A ELISA, respectively. The results were analyzed with Molecular Devices SoftMax Pro® GxP software, version 5.4.1, using a four-parameter algorithm. For each sample, the mean concentration of dilutions within the quantitative range of the ELISA was calculated.

An average concentration of eCry3.1Ab and mCry3A in MZIR098 maize crude extract was determined from six independent extracts and was used to define (1) the amount of microbially produced protein required for subsequent preparation of eCry3.1Ab and mCry3A samples for Western blot analysis, and (2) the amount of microbially produced protein required for subsequent preparation of an eCry3.1Ab and mCry3A mixture for insecticidal activity determination.

### Quantitation of purified trait protein by densitometry

The concentration of eCry3.1Ab and mCry3A in respective purified preparations from MZIR098 maize extract was quantified by SDS-PAGE using calibration curves derived from known concentrations of microbially produced eCry3.1Ab, or mCry3A, respectively. Image Lab software (Bio-Rad Laboratories) was used to estimate concentrations for subsequent Western blot analysis, glycosylation blot analysis, and in-solution protein digestion.

### Western blot analysis

Western blot analysis was used to investigate the intactness and apparent molecular weight of the microbially produced and plant-produced eCry3.1Ab and mCry3A. Aliquots of microbially prepared or plant-derived eCry3.1Ab and mCry3A were prepared in NuPAGE® LDS sample buffer.

In addition, crude extract of nontransgenic maize leaf powder was fortified with either microbially produced eCry3.1Ab or mCry3A, for respective samples that were included in the Western blot analysis to allow for comparison with the plant-produced eCry3.1Ab and mCry3A in the same matrix. These comparisons helped to interpret any plant matrix effect on the immunoreactivity and mobility of each respective insecticidal protein. The molecular weight standard was SeeBlue® Plus2. All samples were subjected to SDS-PAGE under reducing conditions with a NuPAGE® 4–12% Bis–Tris polyacrylamide gradient gel using morpholinoethanesulfonic acid (MES) running buffer (Life Technologies). The protein was transferred to a polyvinylidene fluoride (PVDF) membrane (Life Technologies) via electroblotting. After electroblotting, the membrane was probed with polyclonal goat antibodies capable of detecting eCry3.1Ab and mCry3A protein. Detection was accomplished through binding of secondary polyclonal alkaline-phosphatase-conjugated donkey anti-goat IgG antibodies (Jackson ImmunoResearch Laboratories, Inc.). Visualization was accomplished through the alkaline phosphatase which catalyzed the conversion of the chromogenic substrate solution BCIP®/NBT (Sigma-Aldrich). The Western blot was examined for the presence of intact immunoreactive eCry3.1Ab and mCry3A or other immunoreactive eCry3.1Ab- and mCry3A-derived fragments.

### Peptide mass coverage analysis

Proteolytic peptides for peptide mass coverage analysis were produced using an in-solution protein digestion. The microbially produced eCry3.1Ab and mCry3A, and the purified eCry3.1Ab and mCry3A preparations from MZIR098, were each solubilized in ammonium bicarbonate, pH 8.0, then reduced with 10 mM dithiothreitol (in ammonium bicarbonate, pH 8.0) for 30 min at approximately 50 °C. Each sample was then alkylated in 50 mM iodoacetamide for 30 min at ambient temperature in the dark. For the in-solution protein digestion, the reduced and alkylated protein samples were hydrolyzed separately with three endoproteinases, trypsin, chymotrypsin, and endoproteinase Asp-N (Roche Diagnostics) at approximately 37 °C overnight. The resultant proteolytic peptides were shipped on dry ice to Caprion BioSciences Inc. (Montreal, Quebec, CA) for peptide mass coverage analysis to determine the identity of the respective eCry3.1Ab and mCry3A (both for microbially produced preparations and those purified from MZIR098 maize extracts). Standard instrumentation for liquid chromatography coupled to tandem mass spectrometry (LC-MS/MS) (Ultra-Performance Liquid Chromatography [UPLC®], Waters corporation, Elstree, UK, coupled to a Q Exactive™ mass spectrometer, Thermo Scientific) was used. The in-solution proteolytic peptides were separated using a Waters nanoAcquity UPLC trap column Symmetry C18 (180 µm × 20 mm, 5 µm particle size) and a Waters nanoAcquity UPLC BEH130 C18 analytical column (150 µm × 100 mm, 1.7 µm particle size) equilibrated with 7.5% B (solvent A = 0.2% formic acid in water; solvent B = 0.2% formic acid in acetonitrile). The peptides were resolved using a 7.5–60% B gradient over 37.5 min with a flow rate of 1.8 µL/min and directly subjected to the mass spectrometer. All MS/MS spectra were obtained in data-dependent mode selecting the 12 most intense multiply charged ions per survey scan (400–1800 m/z, mass-to-charge) for higher-energy collisional dissociation (HCD). The survey scans and HCD spectra were acquired at a resolution of 70,000 and 17,500, respectively. Each acquired MS/MS spectrum was submitted to the Mascot search engine (version 2.2.06, Matrix Science, Boston, MA, US) to obtain the peptide identities, searching against a database containing the respective protein amino acid sequence. Only peptides identified with an ion score greater than the Mascot identity threshold were considered truly identified.

### Glycosylation analysis

The microbially produced and MZIR098 maize-produced eCry3.1Ab and mCry3A were analyzed with the Sigma® Glycoprotein Detection Kit to confirm the absence of glycosyl residues. Samples were separated by SDS-PAGE under reducing conditions using a NuPAGE® 4–12% Bis–Tris gel and MES running buffer. Aliquots containing 25 pmol of eCry3.1Ab and mCry3A purified from MZIR098 maize extract, and 25 pmol of the microbially produced eCry3.1Ab and mCry3A, were applied to the gels. Horseradish peroxidase (HRP), a glycosylated protein, was applied to the gels at 25, 10, 5, 2.5, and 1 pmol as a positive control. Soybean trypsin inhibitor (Sigma), a nonglycosylated protein, was applied to the gels at 25 pmol as a negative control. The MW pre-stained standard was SeeBlue® Plus2 standard. Following SDS-PAGE, the proteins were electroblotted onto a nitrocellulose membrane (Life Technologies). While on the membrane, glycan moieties were oxidized using periodic acid, stained with Schiff’s Fuchsin-Sulfite reagent and reduced with sodium metabisulfite. The blot was removed from the reagents and imaged using an optical imager (Bio-Rad Gel Doc XR + Imager).

After the glycosylation analysis was completed, the blots were washed with water and stained using Swift™ Membrane Stain (G-Biosciences, St. Louis, MO, US) to visualize all proteins to verify the appropriate loading of the microbially produced and plant-produced eCry3.1Ab and mCry3A proteins. The blot was imaged using an optical imager (Bio-Rad Gel Doc XR + Imager).

### Insecticidal activity

The Colorado potato beetle (CPB) insect diet (Frontier Scientific, Newark, DE, US) was freshly prepared for a diet incorporation bioassay as described in Graser et al. (2017). The insecticidal activity of a mixture of eCry3.1Ab and mCry3A from (1) MZIR098 maize crude extract, (2) microbially produced eCry3.1Ab and mCry3A, and (3) nontransgenic maize crude extract fortified with a mixture of microbially produced eCry3.1Ab and mCry3A were determined in bioassays against first instar CPB larvae. The microbially produced eCry3.1Ab and mCry3A proteins were each solubilized in 50 mM Tris, 2 mM ethylenediaminetetraacetic acid, pH 10.5 buffer and the appropriate mixture prepared according to the average concentrations determined by the ELISA analyses of MZIR098 maize crude extracts. The microbially produced eCry3.1Ab and mCry3A mixture was then diluted in PBST or nontransgenic maize crude extract which was equivalent in total protein to the MZIR098 maize crude extract for respective bioassay samples, followed by respective dilution series in PBST. MZIR098 maize crude extract was similarly diluted in PBST for respective bioassay samples. Nontransgenic maize crude extract, which was equivalent in total protein to the MZIR098 maize crude extract, as well as PBST buffer alone were included in the bioassays as negative controls. Each treatment dilution series was mixed 1:1 (v/v) with the freshly prepared CPB diet to produce eight diets with final concentrations of 2.84, 2.00, 1.42, 1.00, 0.71, 0.36, 0.18, and 0.09 µg insecticidal protein mixture/ml diet. The two control samples were also mixed 1:1 (v/v) with the freshly prepared CPB diet.

The bioassays were conducted in 24-well Costar culture plates (Corning, Inc., Kennebunk, ME, US) with each well containing one freshly hatched CPB insect larva as described in Graser et al. (2017), except each well contained 200 µl of the respective diet mixture and plates were then stored at 22 ± 5 °C, with a 14 h/10 h light/dark cycle. Plates were placed within the incubator in a non-systematic way, repositioning each successive day to minimize any potential systematic error associated with the environmental condition. An average mortality between the nontransgenic maize crude extract and the PBST buffer alone samples was determined in each independent bioassay. This average control mortality was used during probit analysis of MZIR098 maize crude extract and fortified nontransgenic maize crude extract samples for respective LC_50_ determinations. The mortality of PBST buffer alone was used as the bioassay control during probit analysis of samples containing a mixture of microbially produced eCry3.1Ab and mCry3A. Bioactivity data from three independent bioassays at a 144 h endpoint were combined and used to report total mortality and generate LC_50_ values for each respective treatment.

Means were calculated using Microsoft Office Excel® 2010 software. LC_50_ determination and slope parameters for the bioassays against CPB were calculated using the US EPA Probit Analysis Program, version 1.5.

## Results

### Quantitation of proteins in maize crude extracts

The respective amounts (± SD) of total protein in MZIR098 maize and nontransgenic maize crude extracts, were 6132 (152) and 6480 (315) μg/ml as established by BCA assay (Table S1). For the MZIR098 maize crude extract, the respective concentrations (± SD) of eCry3.1Ab and mCry3A (as determined by ELISA) were 3939 (1137) and 2083 (86) ng/ml, giving a total insecticidal protein concentration of 6022 ng/ml (Table S2). A ratio of eCry3.1Ab to mCry3A in the MZIR098 maize crude extract was therefore estimated to be 1.89 to 1.

### Quantitation of purified trait protein by Densitometry

Following excision from preparative SDS-PAGE gels, gel electro-elution and concentration, the samples were applied to an analytical SDS-PAGE gel. Following staining and destaining, densitometry was performed on separate sample gels containing purified preparations of MZIR098-derived eCry3.1Ab and mCry3A protein. The concentration of insecticidal trait protein purified from MZIR098 maize crude extract was 62 ng/μl and 86 ng/μl, for eCry3.1Ab or mCry3A, respectively. The respective concentrated samples were stored in 25 mM ammonium bicarbonate buffer at -20ºC ± 8ºC until further use in Western blot, glycosylation blot, or peptide mass coverage analyses.

### Western blot analysis

**Fig. 1:**
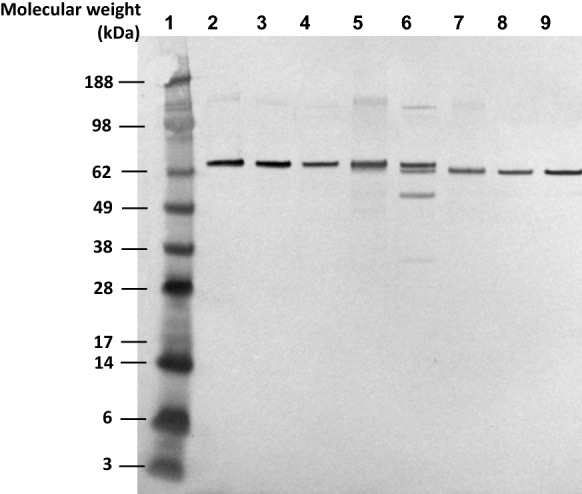
Western blot analysis of microbially and plant-produced eCry3.1Ab and mCry3A. Lane 1: Molecular weight standard; Lanes 2, 3: Microbially produced eCry3.1Ab (7.44 ng eCry3.1Ab); Lane 4: Nontransgenic maize extract (12.2 μg total protein) fortified with microbially produced eCry3.1Ab (7.44 ng eCry3.1Ab); Lane 5: eCry3.1Ab purified preparation from MZIR098 maize extract (7.44 ng eCry3.1Ab); Lane 6: MZIR098 maize crude extract (12.2 μg total protein, 7.44 ng eCry3.1Ab, 3.94 ng mCry3A); Lane 7: mCry3A purified preparation from MZIR098 maize extract (3.94 ng mCry3A); Lane 8: Nontransgenic maize extract (12.2 μg total protein) fortified with microbially produced mCry3A (3.94 ng mCry3A); Lane 9: Microbially produced mCry3A (3.94 ng mCry3A)

Western blot analysis of the microbially produced and plant-produced eCry3.1Ab revealed immunoreactive bands consistent with the respective predicted molecular weights of 74.8 kDa for samples containing microbially produced eCry3.1Ab (Fig. 1, lanes 2, 3 and 4) and 73.7 kDa for samples containing plant-produced eCry3.1Ab (Fig. 1, lanes 5 and 6). The slight difference in molecular weight between the microbially produced eCry3.1Ab and the eCry3.1Ab in the plant-produced samples is expected due to the additional seven amino acids, one methionine and six histidine residues, added to the N-terminus of the microbially produced protein. Western blot analysis also confirmed the predicted molecular weight of 67.7 kDa for samples containing microbially produced mCry3A (Fig. 1, lanes 8, 9) and for samples containing plant-produced mCry3A (Fig. 1, lanes 6 and 7). Two bands of lower molecular weight (ca. 55 kDa, and ca. 35 kDa), were only present in the plant derived sample of MZIR098 maize crude extract (Fig. 1, lane 6); these bands most likely correspond to some degradation of mCry3A as they are consistent with products that can arise from protease activity on Cry3A (Carroll et al. 1989, 1997; Loseva et al. 2002). Western blot analysis also revealed a faint protein band with a molecular weight of approximately 150 kDa (Fig. 1, lanes 2 to 7). This band most likely represents dimers of eCry3.1Ab or mCry3A insecticidal protein because it cross-reacted with the same antibody that detected eCry3.1Ab and mCry3A, was present in samples from different production hosts, and because its apparent molecular weight is consistent with that of two insecticidal protein molecules.

### Peptide mass coverage analysis of eCry3.1Ab and mCry3A

**Fig. 2:**
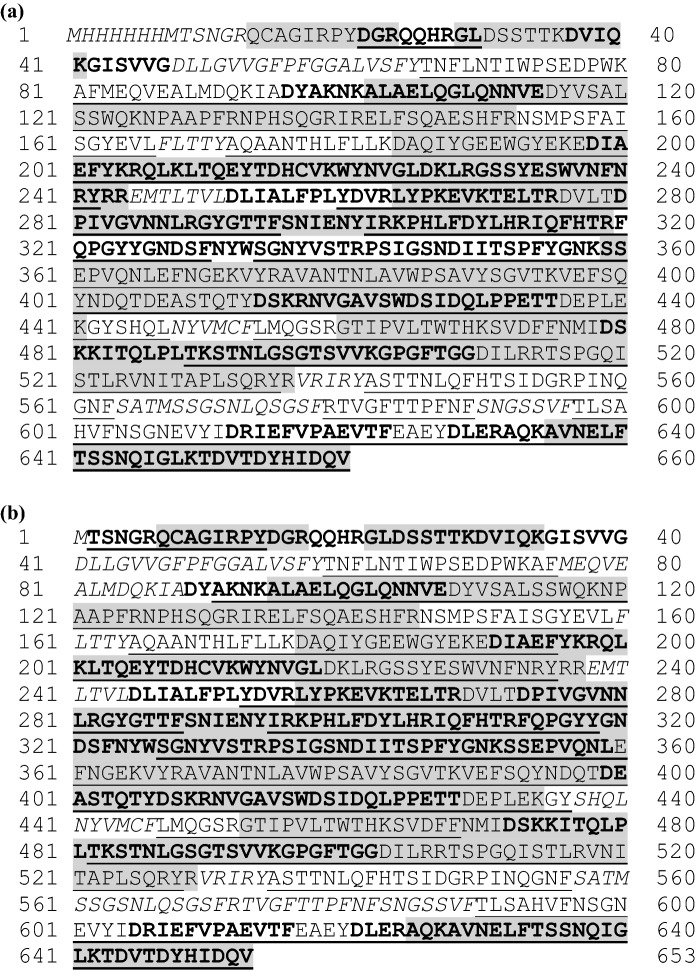
Amino acid sequence coverage map for the **a** microbially produced eCry3.1Ab protein and **b** plant-produced eCry3.1Ab protein. The observed tryptic, chymotryptic, and endoproteinase Asp-N peptides using peptide mass fingerprinting are shaded, underlined, and bolded, respectively, within the deduced amino acid sequence shown. Amino acids italicized were not identified

The collective analysis of the three proteolytic digests for the microbially produced eCry3.1Ab protein resulted in coverage of 88.5% of the total predicted eCry3.1Ab amino acid sequence (Fig. 2a). Evidence for 57.3%, 79.7%, and 43.8% of the eCry3.1Ab protein amino acid sequence coverage was obtained for trypsin, chymotrypsin, and endoproteinase Asp-N, respectively. Similarly, the collective analysis of the three proteolytic digests for the purified eCry3.1Ab preparation from MZIR098 maize extract resulted in coverage of 85.9% of the total predicted eCry3.1Ab amino acid sequence (Fig. 2b). Evidence for 64.3%, 75.0%, and 46.4% of the eCry3.1Ab protein amino acid sequence coverage was obtained for trypsin, chymotrypsin, and endoproteinase Asp-N, respectively.

In addition to the overall amino acid sequence coverage, the N-terminal and C-terminal peptide sequences were evaluated by peptide mass coverage analysis for the microbially produced eCry3.1Ab and the plant-produced eCry3.1Ab protein. The N-terminal peptide was consistent with the predicted sequence for the plant-produced eCry3.1Ab, except for the missing N-terminal methionine (Fig. 2b). The removal of the N-terminal methionine is a common process for many proteins that occurs during translation (Spremulli 2000; Walling 2006). The N-terminal peptide was not observed for the microbially produced eCry3.1Ab protein. However, an intact mass analysis result for the microbially produced eCry3.1Ab protein (74,833.69 Da, Fig. S1) was in agreement with the theoretical mass (74,832.66 Da) predicted from the amino acid sequence M_1_-V_660_ (Fig. 2a), confirming the intended amino acid sequence of the microbially produced protein. The C-terminal peptide from both the microbially produced and plant-produced eCry3.1Ab protein were consistent with the predicted sequences (Fig. 2a, b).

**Fig. 3:**
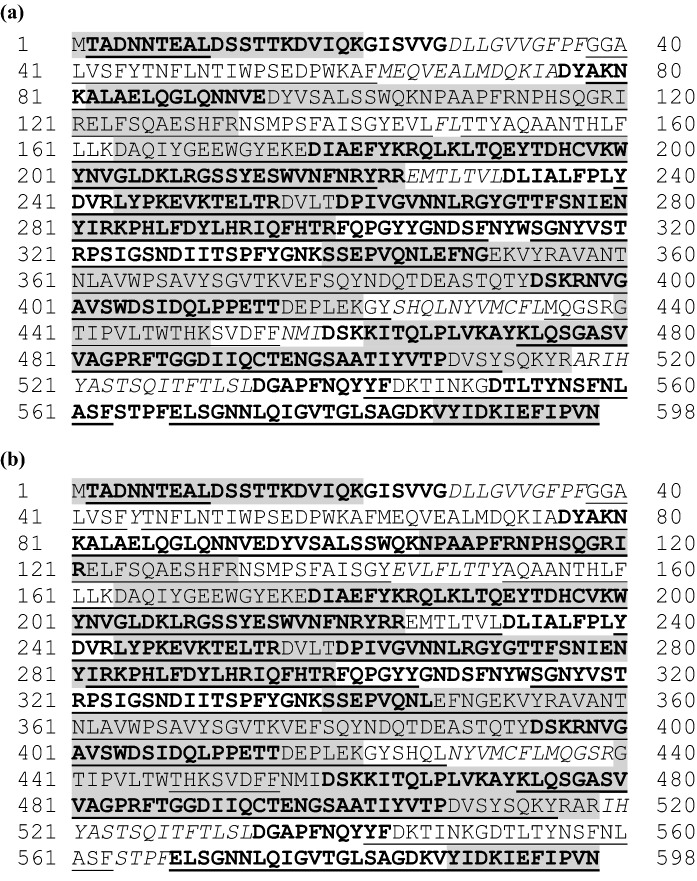
Amino acid sequence coverage map for the **a** microbially produced mCry3A protein and **b** plant-produced mCry3A protein. The observed tryptic, chymotryptic, and endoproteinase Asp-N peptides using peptide mass fingerprinting are shaded, underlined, and bolded, respectively, within the deduced amino acid sequence shown. Amino acids italicized were not identified

The analysis of the microbially produced mCry3A protein resulted in coverage of 89.5% of the total predicted mCry3A amino acid sequence (Fig. 3a). Evidence for 58.5%, 78.8%, and 56.2% of the mCry3A protein amino acid sequence coverage was obtained for trypsin, chymotrypsin, and endoproteinase Asp-N, respectively. Similarly, the collective analysis of the three proteolytic digests for the purified mCry3A preparation from MZIR098 maize extract resulted in coverage of 91.6% of the total predicted mCry3A amino acid sequence (Fig. 3b). Evidence for 56.7%, 78.3%, and 57.2% of the mCry3A protein amino acid sequence coverage was obtained for trypsin, chymotrypsin, and endoproteinase Asp-N, respectively.

In addition to the overall amino acid sequence coverage, the N-terminal and C-terminal peptide sequences were identified by peptide mass coverage analysis for the microbially produced mCry3A protein and the plant-produced mCry3A protein and were consistent with the predicted sequences from both sources (Fig. 3a, b).

### Glycosylation blot analysis

**Fig. 4:**
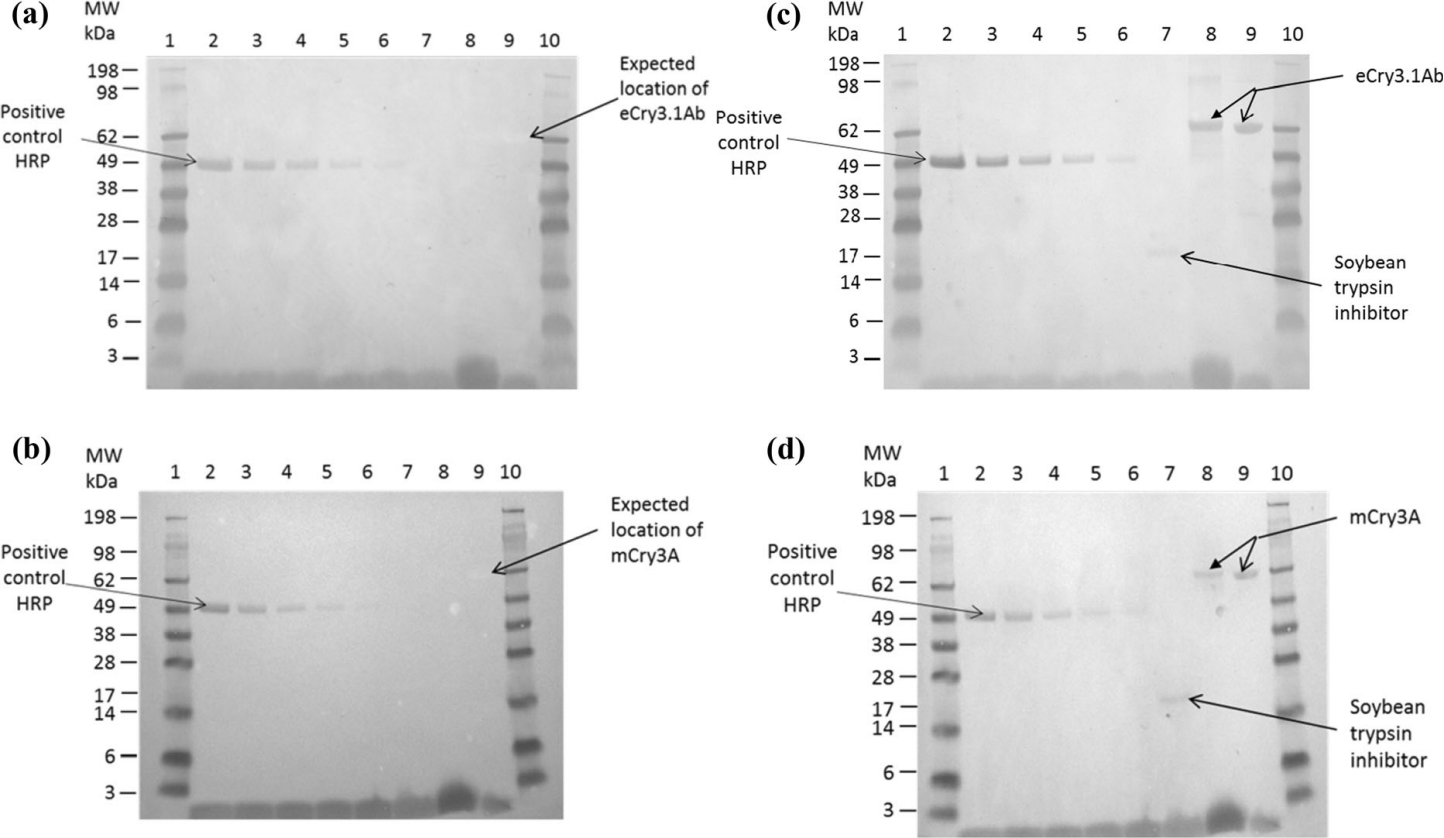
Glycosylation blot analysis of the microbially and plant-produced (**a**) eCry3.1Ab and (**b**) mCry3A. Protein staining to verify sample loading for the microbially and plant-produced (**c**) eCry3.1Ab and (**d**) mCry3A. Lanes 1 and 10: Molecular weight standard; Lanes 2 to 6: HRP, 25, 10, 5, 2.5, or 1 pmol, respectively; Lane 7: Soybean trypsin inhibitor, 25 pmol; Lane 8: 25 pmol eCry3.1Ab (**a** and **c**) or mCry3A (**b** and **d**) purified preparation from MZIR098 maize leaf extract, respectively; Lane 9: 25 pmol microbially produced eCry3.1Ab (**a** and **c**) or mCry3A (**b** and **d**)

The results of the glycosylation analysis of eCry3.1Ab and mCry3A are presented in Fig. 4a and b, respectively. No corresponding stained bands were observed for the microbially produced eCry3.1Ab (Fig. 4a, Lane 9) and mCry3A (Fig. 4b, Lane 9) or plant-produced eCry3.1Ab (Fig. 4a, Lane 8) and mCry3A (Fig. 4b, Lane 8). As expected, the positive control, HRP, generated visible bands consistently decreasing in intensity in correlation with the lower concentrations (Fig. 4a and b, Lanes 2 through 6) confirming the suitability of the assay. Furthermore, the negative control, soybean trypsin inhibitor, did not show visible bands (Fig. 4a and b, Lane 7). The results support the conclusion that neither the microbially produced nor the plant-produced eCry3.1Ab and mCry3A proteins are glycosylated.

### Visualization of eCry3.1Ab and mCry3A on the glycosylation blots by protein staining

To verify appropriate sample load and transfer, the glycosylation blots were stained for protein. The results of the total protein staining of the glycosylation blots using Swift™ Membrane Stain are presented in Fig. 4c and d. The stained blots revealed the presence of bands of similar size and intensity associated with the microbially produced eCry3.1Ab (Fig. 4c, Lane 9) or mCry3A (Fig. 4d, Lane 9) and plant-produced eCry3.1Ab (Fig. 4c, Lane 8) or mCry3A (Fig. 4d, Lane 8) consistent with the predicted MW for the respective eCry3.1Ab and mCry3A proteins. In addition, protein bands corresponding to soybean trypsin inhibitor were visualized in both blots (Fig. 4c and d, Lane 7). These results verified the appropriate and equivalent loading of eCry3.1Ab and mCry3A proteins on the glycosylation blots.

### Determination of insecticidal activity of a mixture of eCry3.1Ab and mCry3A

Following the unique design for the functional equivalence testing, the bioassay samples containing maize extracts were matched in terms of the total protein present and, for those containing insecticidal protein, the amount and ratio of eCry3.1Ab:mCry3A insecticidal protein was also matched. The composite results of the three independent insect bioassays are summarized in Tables 2 and 3. The mixture of eCry3.1Ab:mCry3A insecticidal proteins showed very comparable response ranges (Table 2) and slope parameters which overlapped (Table 3) for treatments from (1) MZIR098 maize crude extract, (2) a mixture of microbially produced eCry3.1Ab and mCry3A, and (3) nontransgenic maize crude extract fortified with a mixture of microbially produced eCry3.1Ab and mCry3A. By comparison, low CPB mortality, of 8% or 4%, was observed in the negative controls using diet made with buffer or nontransgenic maize crude extract, respectively (Table 2). The mixture of eCry3.1Ab:mCry3A insecticidal proteins extracted from MZIR098 maize crude extract gave an LC_50_ of 0.44 µg/ml, with a 95% confidence interval (CI) of 0.33 to 0.56 µg/ml (Table 3). This result was very similar and with overlapping 95% confidence intervals when compared with either the mixture of microbially produced eCry3.1Ab and mCry3A (0.61 µg/ml, with 95% CI = 0.45 to 0.79 µg/ml), or the nontransgenic maize crude extract fortified with a mixture of microbially produced eCry3.1Ab and mCry3A (0.58 µg/ml, with 95% CI = 0.41 to 0.76 µg/ml).

**Table 2:** Insecticidal activity of eCry3.1Ab and mCry3A against CPB for MZIR098 maize crude extract, a mixture of microbially produced eCry3.1Ab and mCry3A, and nontransgenic maize crude extract fortified with a mixture of microbially produced eCry3.1Ab and mCry3A

Concentration (µg/ml of diet)	MZIR098 maize crude extract^a^		Mixture of microbially produced eCry3.1Ab and mCry3A^a^		Fortified nontransgenic maize crude extract^a^	
	CPB dead (no.)	Mortality (%)	CPB dead (no.)	Mortality (%)	CPB dead (no.)	Mortality (%)
0.09	18	25	10^b^	14	17	24
0.18	24	33	20	28	26	36
0.36	34^b^	48	35	49	30	42
0.71	43	60	45	63	41	57
1.00	50	69	47	65	44	61
1.42	61	85	53^b^	75	48	67
2.00	58^b^	82	51	71	59	82
2.84	66	92	57	79	58	81
Buffer control	6	8	–	–	–	–
Nontransgenic extract control	3	4	–	–	–	–

*N* = 72 except as indicated

^a^Ratio of eCry3.1Ab to mCry3A = 1.89:1. Mortality determined at 144 h

^b^*N* = 71

**Table 3:** Estimated 50% lethal concentration for eCry3.1Ab and mCry3A against CPB for MZIR098 maize crude extract, a mixture of microbially produced eCry3.1Ab and mCry3A, and nontransgenic maize crude extract fortified with a mixture of microbially produced eCry3.1Ab and mCry3A

Treatment	LC_50_ (µg/ml of diet)	95% CI (µg/ml of diet)	Slope ± SEM	95% CI
MZIR098 maize crude extract^a^	0.44	0.33–0.56	1.47 ± 0.15	1.17–1.77
Mixture of microbially produced eCry3.1Ab and mCry3A^a^	0.61	0.45–0.79	1.31 ± 0.15	1.02–1.61
Nontransgenic maize crude extract fortified with a mixture of microbially produced eCry3.1Ab and mCry3A^a^	0.58	0.41–0.76	1.18 ± 0.14	0.90–1.46

^a^Ratio of eCry3.1Ab to mCry3A = 1.89:1. Mortality determined at 144 h

In the context of insect bioassays, it is important to consider the relative magnitude of any apparent differences when comparing two given samples. Reproducibility can be a challenge in obtaining estimates of a single parameter (e.g., an LC_50_ obtained by probit analysis) to describe insect bioassay dose-responses (Robertson et al. 2007; Graser and Walters 2016). As indicated by Robertson et al. (2007) the assumptions required by the probit model make the description of potency (LC_50_) “an estimate, not a measurement” … and “sampling error and natural variation will contribute to the appearance of different potency values from the same sample in repeated bioassays at the same time and to different values over time” (Robertson et al. 2007). Due to this inherent variation with activity assays in biological systems, several authors have recommended use of a built-in threshold of at least twofold above the “predicted response” for even considering a result as different from the expected value (Belden et al. 2007; Cedergreen 2014; Rodea-Palomeres et al. 2015). (Note: in the context of this present work, the samples containing insecticidal protein were expected to have equal bioactivity).

A further assessment was made of the activity for the fortified nontransgenic maize crude extract (fortified with a mixture of microbially produced eCry3.1Ab and mCry3A) compared with the MZIR098 maize crude extract. The LC_50_ estimates from the two probit analyses for these samples (which each contained plant matrix) were compared using the ratio method (Robertson and Preisler 1992; Wheeler et al. 2007). The ratio of the two LC_50_ estimates was estimated along with a 95% CI for the ratio. The ratio was estimated as 1.3 (0.58 µg/ml divided by 0.44 µg/ml) with a 95% CI = 0.3 to 5.7. The value of 1 (i.e., estimates the LC_50_ as equal) is well within the confidence interval and therefore in a direct comparison of the two LC_50_ estimates with the ratio method, the LC_50_ estimates are interpreted as similar. In summary, any observed small differences in the estimated toxicity for the MZIR098 maize crude extract as compared with a fortified nontransgenic maize crude extract are within the error ranges for the technique, and these data demonstrate that the microbially produced eCry3.1Ab and mCry3A proteins are functionally equivalent to the proteins produced in MZIR098 maize.

## Discussion

Several challenges were connected to the characterization of the plant insecticidal proteins of MZIR098 and for establishing the equivalence to heterologously produced microbial test substances. Notably, purification/isolation of two plant-produced proteins of similar molecular weight, and with shared sequence elements and corresponding immunogenicity presented a considerable biochemical challenge. Only through the use of SDS-PAGE electro-elution to provide plant-purified proteins for Western blot, peptide mass coverage, and glycosylation blot analyses, could the biochemical equivalence testing be achieved. An additional technical challenge existed in terms of conducting a bioactivity comparison of the microbially produced insecticidal proteins with the plant-produced proteins for the demonstration of functional equivalence. This challenge connected to the higher order insecticidal stacked event is based on proteins with different modes of action but that have the same identified target pest spectrum. The plan to bioassay a defined ratio mixture of the microbially produced eCry3.1Ab and mCry3A proteins was essential to evaluate the functional equivalence using the same insect species. The described strategy demonstrates a first-of-its-kind approach, as compared to other cases of combinations of proteins with distinct target pest spectra that allow for a unique bioassay test organism to be selected for equivalence data generation (e.g., see Raybould et al. 2012).

Areas of consideration in establishing microbially produced protein as a suitable surrogate can include: (1) intactness, (2) immunoreactivity, (3) protein sequence, (4) glycosylation status, and 5) functional activity of the proteins being compared (see Raybould et al. 2013). While these areas are not necessarily given in any specific order, the functional activity determination could be considered the single strongest piece of information to establish equivalence, as it can be seen to directly relate to interpreting results of toxicity and ecotoxicology studies (Raybould et al. 2013). These five areas will be considered for Event MZIR098 below.

1. Western blot was used to assess mobility of eCry3.1Ab and mCry3A proteins and was consistent with the predicted molecular weights for microbially produced and plant-produced eCry3.1Ab (approximately 74.8 and 73.8 kDa, respectively), and the microbially produced and plant-produced mCry3A (approximately 67.7 kDa). These data, indicating that no insertions or modifications of sequence existed between the microbial and plant-derived protein sources for the respective proteins, support the conclusion that the proteins compared were intact.
2. The Western blot results described above also supported a conclusion of similar immunoreactivity, indicating that no changes in protein structure were likely between the sources for the respective proteins.
3. Overall, the peptide mass coverage analysis confirmed the identity of both insecticidal proteins, and supported a conclusion that no (unintended) differences exist in the amino acid sequence between the sources for the respective proteins. The multi in-solution proteolytic digest strategy (using trypsin, chymotrypsin, and endoproteinase AspN), the bottom-up proteomic approach using LC-MS/MS to characterize each protein, and the bioinformatic analysis conducted using Mascot MS/MS ions combined to deliver as complete a map of primary sequence of each protein as possible. This technical approach resulted in a very high percent of sequence coverage ( ≥85.9% coverage of the total predicted amino acid sequence) for each of the proteins, regardless of the starting source material, and confirmed that detected sequence from both sources were consistent with the predicted sequences.
4. A robust and reproducible approach was established to assess the glycosylation status of the eCry3.1Ab and mCry3A proteins. In brief, it consisted of: a) using matched equimolar positive and negative controls and trait protein, b) a dilution series of positive blot controls to demonstrate the range of sensitivity, c) an internal blot confirmation of a “negative stain” result by virtue of the large amount of trait protein present, and 4) addition of a total protein staining step after the glycosylation assessment was completed as further confirmation of appropriate and equivalent sample loading. There was no evidence of post-translational glycosylation of eCry3.1Ab or mCry3A purified from MZIR098 maize leaf material or from the respective microbially produced proteins as shown by blot glycosylation assay. In general, there is a lack of consensus of the predictive value of considering the glycosylation status and any bearing on subsequent allergenicity of proteins (Altmann 2007; Garrido-Arandia et al. 2014; Valenta et al. 2015; Homann et al. 2017). However, demonstrating that the microbially produced and plant-produced proteins are not differentially glycosylated (as was established for both eCry3.1Ab and mCry3A), can further support a conclusion that the two protein sources can be expected to display similar physicochemical properties.
5. ELISA was used to characterize the ratio of the two insecticidal proteins in crude extracts of MZIR098, then this ratio was matched by a sample made up of a microbially produced mixture of the proteins. Bioassay of the matched sample mixtures of eCry3.1Ab:mCry3A insecticidal proteins showed very comparable bioassay response ranges, slope parameters, and overlapping LC_50_ estimates for treatments from 1) MZIR098 maize crude extract, 2) microbially produced eCry3.1Ab and mCry3A, and 3) nontransgenic maize crude extract fortified with a mixture of microbially produced eCry3.1Ab and mCry3A. In addition, a further assessment of the respective LC_50_ estimates for the fortified nontransgenic maize crude extract compared with the MZIR098 maize crude extract (using the ratio method; Robertson and Preisler 1992; Wheeler et al. 2007) also interpreted the bioactivity as similar. These results support the conclusion that there is no detectable difference in functional activity between the sources for the insecticidal proteins.

## Conclusion

The results presented here strongly support that the microbially produced eCry3.1Ab and mCry3A are suitable surrogates for those proteins as derived from event MZIR098 maize for the purposes of risk assessment. Several novel approaches for protein purification and testing were required to establish the biochemical and functional equivalence. Precise and rigorous methodology was used to obtain plant-purified trait proteins appropriate for the in vitro biochemical equivalence work, and a first-of-its-kind equivalence testing bioassay addressed specific needs of a higher order insecticidal stacked event which is based on proteins with different modes of action but that have the same target pest spectrum.

The purification and testing approaches were complemented by a strategy to obtain high quality peptide mass coverage analysis data, and a glycosylation blot experimental design that supported delivery of robust and reproducible data. In the future, consideration of these strategies that were used for event MZIR098 may be applicable for other molecular stack equivalence testing needs.

## Electronic supplementary material

Below is the link to the electronic supplementary material.

Supplementary file1 (DOCX 51 kb)

Supplementary file2 (DOCX 30 kb)

Supplementary file3 (DOCX 618 kb)

## Abbreviations

*Bt*: Bacillus thuringiensis
CPB: Colorado potato beetle
CRW: Corn rootworm
IRM: Insect resistance management
LC-MS/MS: Liquid chromatography coupled to tandem mass spectroscopy
PBST: Phosphate buffered saline, pH 7.4 with 0.05% Tween® 20
