# Supplementary material for: Meeting technical challenges for protein characterization and surrogate equivalence studies that resulted from insecticidal protein co-expression in maize event MZIR098
Source: Transgenic Res. 2019 Nov 28;29(1):109–24. doi: 10.1007/s11248-019-00183-w (PMC7000486; doi:10.1007/s11248-019-00183-w)
Supplement: Supplementary file 1 — Supplementary file1 (DOCX 51 kb) [file 11248_2019_183_MOESM1_ESM.docx]

**Table S1.**  Total protein determination for lyophilized maize leaf tissue crude extracts.

**Overall**

**Overall**

**Sample**

**Sample**

**Mean Total Protein**

**Mean**

**S.D.**

**Assay date**

**Sample**

**(in μg/ml)**

**(in μg/ml)**

**μ**

**(in μg/ml)**

**μ**

2/17/2014

NEG 1

a

6263

2/17/2014

NEG 2

6149

2/18/2014

NEG 1

6316

2/18/2014

NEG 2

6075

6132

152

2/19/2014

NEG 1

6099

2/19/2014

NEG 2

5887

2/17/2014

POS 1

b

6482

2/17/2014

POS 2

6947

2/18/2014

POS 1

6464

6480

315

2/18/2014

POS 2

6661

2/19/2014

POS 1

6017

2/19/2014

POS 2

6308

a

NEG = Nontransgenic lyophilized maize leaf

b

POS = MZIR098 lyophilized maize leaf
