# Supplementary material for: Meeting technical challenges for protein characterization and surrogate equivalence studies that resulted from insecticidal protein co-expression in maize event MZIR098
Source: Transgenic Res. 2019 Nov 28;29(1):109–24. doi: 10.1007/s11248-019-00183-w (PMC7000486; doi:10.1007/s11248-019-00183-w)
Supplement: Supplementary file 2 — Supplementary file2 (DOCX 30 kb) [file 11248_2019_183_MOESM2_ESM.docx]

**Table S2.** Quantitation of eCry3.1Ab and mCry3A in lyophilized MZIR098 maize leaf crude extracts by ELISA assay.
