# Supplementary material for: Meeting technical challenges for protein characterization and surrogate equivalence studies that resulted from insecticidal protein co-expression in maize event MZIR098
Source: Transgenic Res. 2019 Nov 28;29(1):109–24. doi: 10.1007/s11248-019-00183-w (PMC7000486; doi:10.1007/s11248-019-00183-w)
Supplement: Supplementary file 3 — Supplementary file3 (DOCX 618 kb) [file 11248_2019_183_MOESM3_ESM.docx]

**[M + H]^+^**

**74833.69**

**100**

**Percent**

**0**

**Mass**

**Figure S1.** Intact mass measurement of microbially produced eCry3.1Ab protein. All intact mass measurements were made using a quadrupole time-of-flight mass spectrometer (Q-ToF Premier, Waters corporation, Elstree, UK), with samples delivered via an Agilent HP 1100 HPLC system (Agilent Technologies, Waldbronn, DE). The resultant multiply charged mass spectrum was deconvoluted to produce a spectrum on a mass scale, as shown. The measured mass of microbially produced eCry3.1Ab [M+H]^+^ was found to be 74833.69 Da.
